# Prediction of Cardiac Resynchronization Therapy Response Using Quantitative Gated Myocardial Perfusion Imaging

**DOI:** 10.19102/icrm.2023.14014

**Published:** 2023-01-15

**Authors:** Yasser A. Abdellatif, Ahmad M. Onsy, Salah Eldin H. Eldemerdash, Mona M. Rayan, Hesham M. Abu Shouk, Haitham A. Badran

**Affiliations:** ^1^Department of Cardiology, Faculty of Medicine, Ain Shams University, Cairo, Egypt; ^2^Department of Cardiology, Faculty of Medicine, 6th of October University, 6th of October City, Egypt

**Keywords:** Cardiac resynchronization therapy, gated single-photon emission computed tomography, heart failure, myocardial infusion, myocardial perfusion imaging

## Abstract

Conventional selection criteria are not consistently able to discriminate between responders and non-responders to cardiac resynchronization therapy (CRT). The objective of this study was to evaluate the usefulness of quantitative gated single-photon emission computed tomography (SPECT) in predicting the response to CRT. This prospective cross-sectional study included 25 patients with advanced congestive heart failure who underwent quantitative gated SPECT before and after CRT implantation. Patients with the left ventricular (LV) lead positioned at the latest activation segment away from the scar had a significantly higher chance of responding than those with the lead positioned at a different area. Responders were likely to have a phase standard deviation (PSD) value of >33°, with 86.6% sensitivity and 90% specificity, and a phase histogram bandwidth (PHB) value of >153°, with 100% sensitivity and 80% specificity. Quantitative gated SPECT can help refine patient selection for CRT implantation, using PSD and PHB cutoff points, in addition to guiding the positioning of the LV lead.

## Introduction

Cardiac resynchronization therapy (CRT) is an established therapeutic option for patients with drug-refractory advanced heart failure and ventricular conduction delay.^1^ However, around 30% of patients selected using current selection criteria according to contemporary guidelines do not respond to CRT, which is a serious limitation of this therapy.^2^

Possible explanations for the lack of response to CRT include that a wide QRS complex may reflect interventricular rather than intraventricular dyssynchrony and the QRS duration is indirectly correlated with mechanical dyssynchrony.^3^ Therefore, the assessment of mechanical dyssynchrony may be a better predictor of response to CRT than QRS duration.^4^

Mechanical dyssynchrony can be identified using various myocardial imaging techniques, including echocardiographic tissue Doppler imaging, strain (rate) imaging, speckle tracking or 3-dimensionally derived parameters, magnetic resonance imaging, and nuclear imaging using single-photon emission computed tomography (SPECT).^5^

Gated SPECT is a widely available technique that enables both perfusion and left ventricular (LV) functional parameters to be assessed with low interobserver and intraobserver variability. Phase analysis using gated SPECT was recently evaluated for the assessment of LV dyssynchrony, and a quantitative gated SPECT (QGS) algorithm (from Cedars-Sinai) has been used to provide quantitative parameters for the assessment of LV mechanical dyssynchrony.^6^

This study sought to explore the clinical value of QGS in predicting the response to CRT.

## Patients and methods

### Patients

The present study included 30 patients with advanced congestive heart failure who were eligible for CRT implantation according to the European Society of Cardiology guidelines of pacing released in 2013; patients with symptomatic heart failure despite optimal pharmacological therapy, New York Heart Association (NYHA) functional class III, and ambulatory class IV; and patients with a left ventricular ejection fraction (LVEF) of ≤35%, sinus rhythm, and a QRS duration of ≥120 ms for left bundle branch block (LBBB) morphology or ≥150 ms for non-LBBB morphology.

Patients with a life expectancy of <1 year, pregnant women, patients with atrial fibrillation, and patients with previously implanted pacemakers were excluded.

### Ethical considerations

This study was approved by the Ain Shams University ethical committee and was performed in accordance with the ethical standards laid down in the 1964 Declaration of Helsinki and its later amendments or comparable ethical standards. Informed consent was obtained from all patients.

### Prior to cardiac resynchronization therapy implantation

Data collection was completed for all patients, including history-taking with an emphasis on age, sex, risk factors (such as diabetes mellitus, hypertension, and renal impairment), and a history of previous events (including previous acute coronary syndromes or coronary angiography). Patients were asked about heart failure symptoms, their functional class according to the NYHA classification, and history of current heart failure medications. A questionnaire was also given to patients to examine their quality of life (QOL) using the Arabic version of the Minnesota Living with Heart Failure questionnaire (MLHFQ). The QRS duration was calculated for all patients using 12-lead electrocardiography (ECG), and the QRS morphology was recorded.

### Technetium-99m sestamibi gated single-photon emission computed tomography imaging

All eligible patients underwent resting ECG gated SPECT with technetium (Tc)-99m sestamibi at baseline prior to CRT implantation and at 6 months post-CRT implantation to determine global systolic synchrony parameters, the segment with latest mechanical activation, the extent of perfusion defect, and scar burden; the estimation of LVEF and LV volumes (end-diastolic volume [LVEDV] and end-systolic volume [LVESV]) was also carried out.

The resting images were acquired 1 h after intravenous injection of 20–30 mCi of Tc-99m sestamibi using a dual-headed cardiac dedicated gamma cameral system (Philips Medical Systems, Eindhoven, the Netherlands) equipped with a low-energy high-resolution parallel hole collimator operating in continuous rotation around 360° according to the local protocol of the nuclear cardiology laboratory. An ECG R-wave detector was used to provide gating to acquire 8 emission frames per cardiac cycle.^3^

Visual and semiquantitative analyses were used to obtain a score to represent perfusion for each of the 17 segments of the myocardium based on 3 short-axis slices and a representative long-axis slice to depict the apex. Each segment was scored according to the degree of radiotracer uptake. The defect size was expressed as a percentage of the LV, as previously validated in a phantom model.^7^ The presence of a large total perfusion defect encompassing ≥30% of the LV was diagnosed as ischemic cardiomyopathy.^8^

Automated computer-generated LV volumes and LVEF were obtained from short-axis slices of electrocardiographically gated rest myocardial perfusion SPECT datasets using the AutoQUANT (QGS) software program (Media Cybernetics, Inc., Rockville, MD, USA).

Reconstructed images were analyzed using a modified version of the QGS algorithm (Cedars-Sinai Medical Center) that provides quantitative parameters for the assessment of LV dyssynchrony. In the present study, a histogram and polar map were constructed from the phase value for the entire LV. The phase standard deviation (PSD) and phase histogram bandwidth (PHB) were calculated and examined as the global dyssynchrony parameters, and the segment with the latest mechanical activation was identified.^3^

### Cardiac resynchronization therapy device implantation

**Figure 1:**
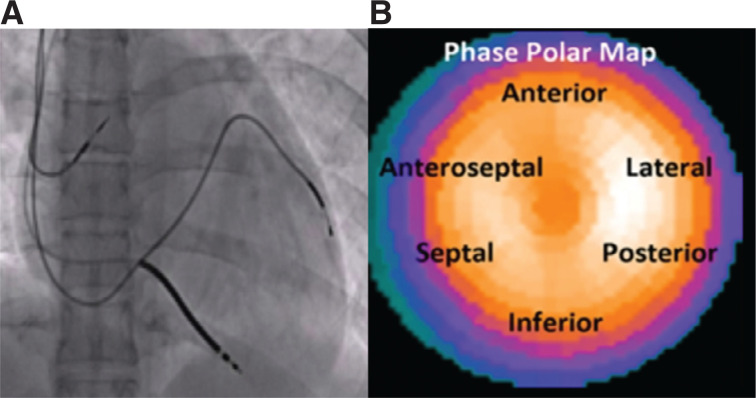
Relation of the left ventricular lead positioned in the lateral cardiac region on fluoroscopy **(A)** and the region of latest mechanical activation located on the phase polar map using the 6-segment model^9^ **(B)**.

All patients underwent CRT implantation, and all leads were implanted transvenously by physicians who were blinded to the results of the SPECT examination. No intraoperative hemodynamic evaluation was performed. The pacing leads were connected to a CRT implantable device (from Biotronic [Berlin, Germany], Medtronic [Medtronic, Minneapolis, MN, USA], Abbott [Chicago, IL, USA], or Boston Scientific [Marlborough, MA, USA]). Then, after CRT implantation, biplane fluoroscopy was performed using orthogonal views (left anterior oblique at 60° and right anterior oblique at 30°). The images were analyzed to determine the anatomic location of the LV lead using the 6-segment model **(Figure 1)**.^9^

The LV lead position was determined using fluoroscopy and classified retrospectively according to the results of the SPECT analysis to be concordant or discordant with the site of the most-delayed mechanical activation determined prior to implantation. The lead was considered concordant when it was found to be adjacent to the latest contracting segment of the LV assessed using SPECT. On the other hand, the LV lead was considered discordant if it was placed in any other coronary sinus tributary that was not adjacent to the latest contracting LV segment.

### Post-implantation follow-up

Patients were assessed 6 months after CRT implantation for the following: NYHA functional class; QOL, using a questionnaire according to the MLHFQ; and QGS to determine LV volumes, LVEF, and global dyssynchrony indices to define the response to CRT.

Responders were defined as patients with a ≥5% improvement in LVEF as well as an improvement of ≥1 NYHA class, whereas non-responders were defined as patients who showed no improvement or a <5% improvement in LVEF and no improvement in NYHA class.^10^

### Data management and statistics

Data were coded and entered into SPSS version 22 (IBM Corporation, Armonk, NY, USA) for analysis. Qualitative data were presented as numbers and percentages. The chi-squared test was used to compare qualitative data, while Fisher’s exact test was only used when the expected count in any cell was <5. Quantitative data are presented as means, standard deviations, and ranges. Comparisons between 2 groups with quantitative data were performed using an independent *t* test when the data were parametric or the Mann–Whitney test when the data were non-parametric, and comparisons between paired data were performed using a paired *t* test. Pearson and Spearman correlation coefficients were used to assess the relationships between 2 parameters in the same group. Logistic regression analysis was used to assess predictors of CRT response after implantation.

Receiver-operating characteristic (ROC) curves were constructed to determine cutoff values for PSD and PHB. The confidence interval was set to 95%, and the margin of error accepted was set to 5%. *P* ≤ .05 was considered statistically significant.

## Results

Thirty patients were enrolled in this study; however, 2 patients died and contact was lost with 3 patients. Therefore, a total of 25 patients fulfilled the inclusion criteria and completed the 6-month follow-up assessments. The mean age was 49 ± 9 years, with 56% of the population being male and 44% of cases having an ischemic etiology. The distribution of risk factors is shown in **Table 1**. The mean MLHFQ score was 68 ± 15 points and 68% of cases were NYHA class III, whereas 32% were NYHA class IV. The mean QRS duration was 156 ± 12 ms with 56% of cases having an LBBB morphology. The mean echocardiographic-derived LVEF was 21.6% ± 5.2%.

These 25 patients were subdivided according to the criteria for CRT response into 2 groups: responders (n = 15) and non-responders (n = 10). Both groups were comparable in terms of demographic data and risk factor distribution. The assessment of symptoms and QOL revealed that the responders showed significant degrees of NYHA class III and IV compared to the non-responders, whereas there were no significant differences in QOL **(Table 1)**. ECG findings revealed that the incidence of LBBB was significantly higher in the responder group, whereas the QRS duration showed no significant difference between the 2 groups.

A comparison of the myocardial perfusion data of both groups showed that the responder group had significantly smaller LV volumes (LVEDV and LVESV) at baseline. The responder group also presented a greater baseline degree of mechanical dyssynchrony as determined using phase analysis. On the other hand, patients in the responder group had a significantly smaller perfusion defect. In the responder group, there was also a significantly higher percentage of patients with coronary sinus lead position concordant with the area of latest activation and not adjacent to scar tissue **(Table 1)**.

**Figure 2:**
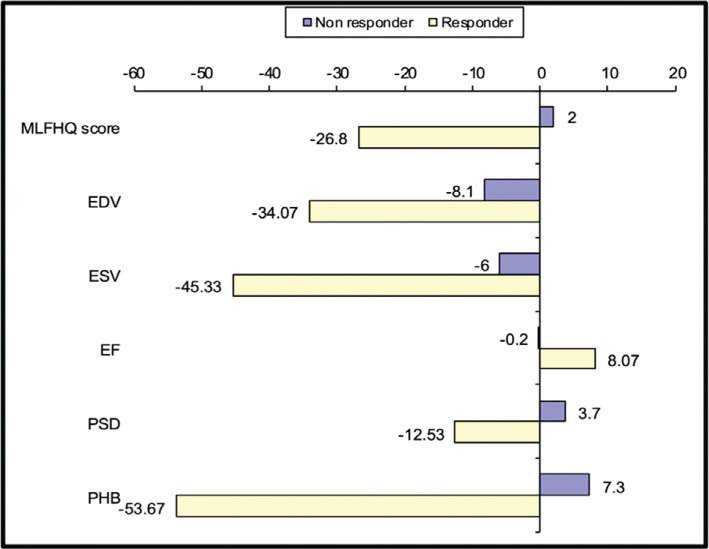
Comparison between the responder and non-responder groups regarding the 6-month change in Minnesota Living with Heart Failure Questionnaire score and single-photon emission computed tomography parameters after cardiac resynchronization therapy device implantation. *Abbreviations:* EDV, end-diastolic volume; EF, ejection fraction; ESV, end-systolic volume; MLHFQ, Minnesota Living with Heart Failure Questionnaire; PHB, phase histogram bandwidth; PSD, phase standard deviation.

At the 6-month follow-up, both groups were compared using MLHFQ scores and SPECT parameters. The responder group showed significant improvements in the MLHFQ score compared to the non-responder group (*P* < .001). Considering SPECT parameters, the responder group showed significant improvements in the dyssynchrony indices (*P* < .001) and significant reverse remodeling with improvements in LVEF **(Table 2, Figure 2)**.

**Figure 3:**
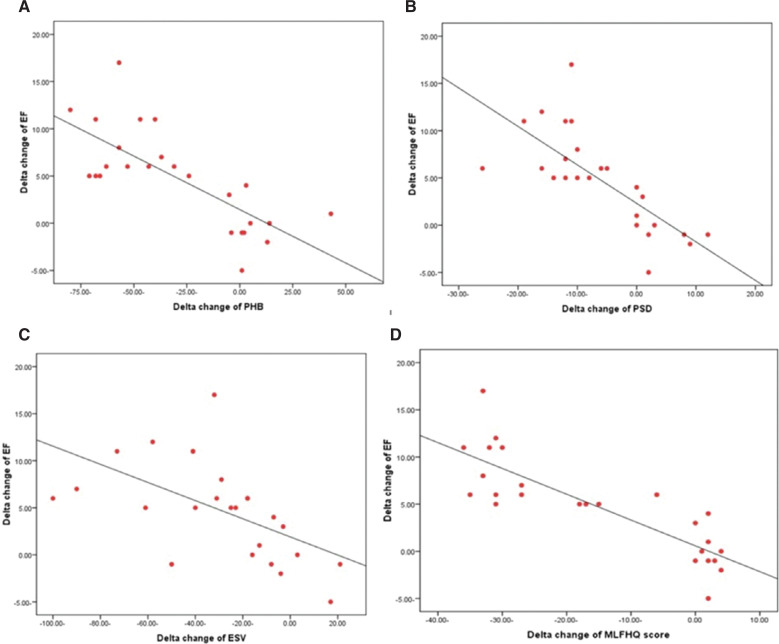
**A:** Correlation between the 6-month change in phase histogram bandwidth and left ventricular ejection fraction (LVEF) (r = −0.725, *P* < .001). **B:** Correlation between the 6-month change in PSD and LVEF (r = −0.815, *P* < .001). **C:** Correlation between the 6-month change in left ventricular end-systolic volume and LVEF (r = −0.699, *P* < .001). **D:** Correlation between the 6-month change in Minnesota Living with Heart Failure Questionnaire score and LVEF (r = −0.871, *P* < .001). *Abbreviations:* ESV, end-systolic volume; MLHFQ, Minnesota Living with Heart Failure Questionnaire; PHB, phase histogram bandwidth; PSD, phase standard deviation.

Considering the change in LVEF at 6 months, both the change in MLHFQ score and the change in mechanical dyssynchrony indices (PSD and PHB) negatively correlated with the change in LVEF. This implies that a lower MLHFQ score (better QOL) correlates with improved LVEF, and the lower the dyssynchrony indices (better synchrony), the greater the improvement in LVEF. On the other hand, the baseline QRS duration was not significantly correlated with the change in LVEF **(Table 3, Figure 3)**.

In the present study, univariate regression analysis for predictors of CRT response revealed that non-ischemic etiology and baseline NYHA (III rather than IV) are predictors of such. Baseline SPECT-derived parameters that predict CRT response include smaller baseline LV volumes (LVEDV and LVESV), higher dyssynchrony indices (PSD and PHB), lower total perfusion defects, and placing the LV lead adjacent to the SPECT-determined latest activation and away from the scar **(Table 4)**.

**Figure 4:**
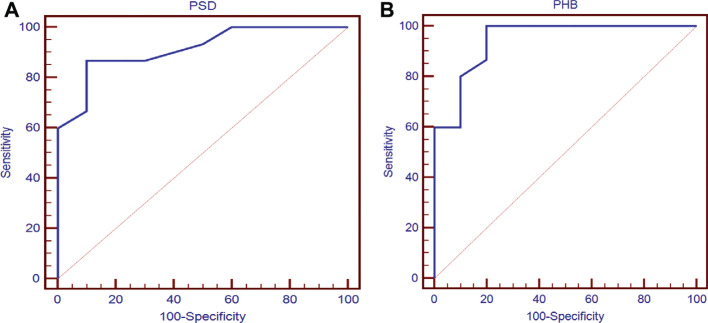
Receiver-operating characteristic curve analysis of gated myocardial perfusion single-photon emission computed tomography for phase standard deviation **(A)** and phase histogram bandwidth **(B)** for prediction of the response to cardiac resynchronization therapy. *Abbreviations:* PHB, phase histogram bandwidth; PSD, phase standard deviation.

In the present study, ROC analysis was performed to determine the cutoff value of dyssynchrony indices to predict CRT response. A cutoff value for PSD of 33°, with a sensitivity of 86.6% and specificity of 90% (area under the ROC curve [AUC], 0.913), was determined. Therefore, patients with PSD > 33° were likely to be responders. A cutoff value for PHB of 153°, with a sensitivity of 100% and a specificity of 80% (AUC, 0.943), was also determined, and patients with PHB values > 153° were likely to be responders **(Table 5, Figure 4)**.

## Discussion

An important aspect in the management of heart failure is to obtain and maintain optimal electrical activation of the cardiac chambers as well as atrioventricular synchrony.^11^ Current guidelines for biventricular pacing adopt the electrographic criteria in terms of QRS duration and morphology that reflect electrical rather than mechanical dyssynchrony. However, based on these criteria, 30% of patients undergoing CRT implantation are non-responders. Efforts have been made to decrease non-responder rates, including improving patient selection, optimizing the correction of electrical and mechanical dyssynchrony, and improving postimplant care.^12^ Echocardiographic techniques, such as tissue Doppler and strain rate imaging, have been used to identify mechanical dyssynchrony. However, these methods have some disadvantages as they lack reproducibility and the ability to differentiate between responders and non-responders to CRT.^13^

SPECT has been used to determine dyssynchrony indices using phase analysis, which has been shown to be reproducible and has the potential to predict the response to CRT.^3^ Thus, using reproducible SPECT dyssynchrony indices to address the mechanical dyssynchrony has been suggested to refine the criteria for CRT response. The assessment of LV mechanical dyssynchrony using phase analysis of gated myocardial perfusion SPECT was feasible in all patients using 2 selected indices—namely, PSD and PHB. As CRT is indicated to synchronize ventricular pacing, CRT will fail to produce tangible results if the baseline dyssynchrony is below a certain threshold. We explored the potential of baseline dyssynchrony indices to predict the response to CRT. In the present study, a cutoff point of 33° for PSD was detected, with a sensitivity of 86.6% and specificity of 90%, whereas a cutoff point for PHB of 153° was detected, with a sensitivity of 100% and specificity of 80%. These results are close to those from Azizian et al., who reported cutoff points of 21° for PSD and 112° for PHB, respectively.^14^ In addition, it was demonstrated that the improvement in mechanical dyssynchrony indices significantly correlated with the improvement in LVEF, a result which is quite similar to that highlighted in the VISION-CRT study.^15^

SPECT imaging is not only capable of dyssynchrony evaluation but also allows simultaneous assessment of LV perfusion and function. In addition, it can assess myocardial scar location and severity for optimizing CRT in patients with heart failure.^15^ Prior to CRT, it seems logical to select the position of the LV lead insertion in the coronary sinus vein as one adjacent to the latest electromechanical activation–viable segment to achieve optimal resynchronization.^16^ The priority is to achieve a stable LV lead position with a suitable threshold in the absence of diaphragmatic pacing in the posterolateral area. All LV leads were positioned in the mid-LV segment in the right anterior oblique projection to avoid apical insertion of the LV lead, as the MADIT-CRT study showed that the apical position is less effective and even harmful, increasing the risk of death.^17^ In the present study, positioning the LV lead concordant with the latest activation segment was a predictor of CRT response. These results agree with those concluded in the study by Boogers et al. using SPECT and similarly in the TARGET trial with speckle-tracking site analysis.^18,19^ Nevertheless, in the VISION-CRT study using SPECT, the investigators failed to show this association that was possibly attributed to a lack of operator blinding to the echo information of sites of scar that could have influenced the lead positioning decision.^15^ It is also noteworthy that reliable echocardiographic measurements require expertise in order to obtain consistent and reproducible results.

Extensive scar tissue in the LV may hinder the response to CRT. Accordingly, patients with extensive scarring in the LV were less likely to be responders to CRT.^20,21^ Additionally, patients with non-ischemic cardiomyopathy are more likely to be responders than those with ischemic cardiomyopathy.^22^ In the present study, the total defect size that reflects scar burden was significantly larger in the non-responder group. This finding is supported by Adelstein et al. and Sciagrà et al., who reported that patients with extensive resting perfusion defects had a limited clinical response to CRT.^21,23^ This may be explained by the direct correlation between LV scar and LV dyssynchrony. Samad et al. used SPECT imaging to show that the presence of LV scar and its extent were independent predictors of mechanical dyssynchrony.^24^ In addition, Ludwig et al. proposed that PSD values were increased by the presence of scar tissue.^25^

In the present study, the relationship between the scar and LV lead position had a significant impact on CRT response. Within the responder group, only 27% of patients had the LV lead concordant to the scarred area, while 80% of patients in the non-responder group had the lead concordant to the scarred area. This result was similar to the findings reported by Leyva et al. and Bleeker et al., who found that placing the LV lead away from the scar tissue gave clinically better results after implantation of CRT. This could be due to the fact that scar tissue could not respond or contract compared to non-scar tissue; therefore, placing the LV lead adjacent to scar tissue would result in an impaired response.^26,27^

It is noteworthy that other factors that influence the CRT response were demonstrated in the study. The responder group had a significantly higher proportion of female patients, NYHA III (rather than IV), non-ischemic etiology, LBBB morphology, and baseline smaller LV volumes. The higher female sex prevalence in the present study is consistent with the findings reported by Cheng et al., which is possibly explained by the greater rates of non-ischemic etiology and deaths from pump failure rather than sudden cardiac death in female patients.^28–30^ In addition, a lower response in patients with NYHA class IV is similar to that reported by De Sisti et al., which could be attributed to the more adverse remodeling and higher baseline dyssynchrony in patients with NYHA class IV.^31^ The presence of LBBB morphology has been shown to be significantly higher in the responder group, which is commensurate with the guideline recommendations. In the present study, responders showed a significantly smaller heart (lower EDV and ESV) at baseline, which is supported by the findings reported by Azizian et al., who found that responders showed less dilatation in the LV than non-responders, although the difference was not statistically significant.^14^ In addition, significant reverse remodeling was observed in the responder group after CRT implantation compared to the non-responder group, consistent with the results of the study by Boogers et al.^18^

This study has some limitations that need to be reinforced by more specific larger studies in order to contribute to clinical practice. One of the limitations is the unfeasible application of multivariate analysis due to the small number of patients enrolled.

## Conclusion

In conclusion, SPECT represents a “one-stop shop” non-invasive imaging modality that has the potential to refine patient selection for CRT implantation and predict and optimize the CRT response. It not only enables assessment of mechanical dyssynchrony with reproducible indices but also allows for simultaneous assessment of LV perfusion and function. In addition, it can assess myocardial scar location and severity and guide placing of the LV lead adjacent to the gated SPECT-derived latest activation segment and away from the scarred area to achieve a better response.

## Figures and Tables

**Table 1:** Baseline Characteristics of the Study Group

Baseline	Study Population (n = 25)	Responders (n = 15)	Non-responders (n = 10)	*P* value
Demographic data				
Age, mean ± SD	48.76 ± 8.82	46.67 ± 9.03	51.90 ± 7.89	.150
Sex (male)	14 (56%)	6 (40.0%)	8 (80.0%)	.048
Risk factors				
Ischemic heart disease	11 (44%)	4 (26.7%)	7 (70%)	.032
Hypertension	15 (60%)	8(53.3%)	7 (70%)	.405
Diabetes mellitus	10 (40%)	5 (33.3%)	5 (50%)	.405
Obesity	13 (52%)	6 (40%)	7 (70%)	.141
Dyslipidemia	14 (56%)	7 (46.7%)	7 (46.7%)	.250
Chronic kidney disease	6 (24%)	2 (13.3%)	4 (40%)	.126
Clinical assessment				
NYHA class III	17 (68%)	14 (93.3%)	3 (30.0%)	.001
NYHA class IV	8 (32%)	1 (6.7%)	7 (70.0%)	
MLHFQ score, mean ± SD	69.9±16.86	66.07 ± 13.99	69.90 ± 16.86	.542
ECG and echocardiography				
QRS morphology LBBB	14 (56%)	12 (80.0%)	2 (20.0%)	.003
QRS duration, mean ± SD	155.6 ± 11.58	154.67 ± 13.02	157.00 ± 9.49	.632
LVEF Simpson’s, mean ± SD	21.6 ± 5.21	22.93 ± 4.71	19.60 ± 5.52	.119
Baseline gated SPECT parameters				
EDV, mean ± SD	301.12 ± 80.51	265.07 ± 79.77	355.20 ± 44.39	.004
ESV, mean ± SD	243.32 ± 65.04	218.87 ± 66.03	280.00 ± 44.81	.009
LVEF, mean ± SD	19.04 ± 6.39	21.07 ± 6.67	16.00 ± 4.74	.050
PSD, mean ± SD	36.32 ± 9.91	41.67 ± 8.59	28.30 ± 5.27	<.001
PHB, mean ± SD	173.04 ± 35.44	194.20 ± 24.04	141.30 ± 24.16	<.001
Perfusion defect (%), median (IQR)	19 (13–25)	14 (10–16)	30 (20–35)	<.001
Lead position and its concordance with latest activation segment and scar				
Lead position concordance with latest activation (concordant)	13 (52%)	12 (80.0%)	4 (40.0%)	.041
Scar concordance with lead position (concordant)	12 (48%)	4 (26.7%)	8 (80.0%)	.009

*Abbreviations:* ECG, electrocardiogram; EDV, end-diastolic volume; ESV, end-systolic volume; LBBB, left bundle branch block; LVEF, left ventricular ejection fraction; IQR, interquartile range; MLHFQ, Minnesota Living with Heart Failure Questionnaire; NYHA, New York Heart Association; PHB, phase histogram bandwidth; PSD, phase standard deviation; SD, standard deviation; SPECT, single-photon emission computed tomography.

**Table 2:** Comparison Between Study Groups of the 6-month Change in Minnesota Living with Heart Failure Questionnaire and Single-photon Emission Computed Tomography Parameters After Cardiac Resynchronization Therapy Device Implantation

	Responder Group (n = 15)	Non-responder Group (n = 10)	Test Value	*P* value
MLHFQ score	−26.80 ± 8.72	2.00 ± 1.41	10.283	<.001
LVEDV	−34.07 ± 32.99	−8.10 ± 20.83	2.205	.038
LVESV	−45.33 ± 25.74	−6.00 ± 19.56	4.097	<.001
LVEF	8.07 ± 3.56	−0.20 ± 2.53	−6.341	<.001
PSD	−12.53 ± 5.26	3.70 ± 4.35	8.074	<.001
PHB	−53.67 ± 16.22	7.30 ± 13.99	9.705	<.001

*Abbreviations:* CRT, cardiac resynchronization therapy; LVEDV, left ventricular end-diastolic volume; LVEF, left ventricular ejection fraction; LVESV, left ventricular end-systolic volume; MLHFQ, Minnesota Living with Heart Failure Questionnaire; PSD, phase standard deviation; PHB, phase histogram bandwidth.

**Table 3:** Correlation Between Baseline QRS Duration and the 6-month Changes in Studied Parameters and Left Ventricular Ejection Fraction

	Change in LVEF	
	r	*P* value
Baseline QRS duration	0.039	.852
Change in MLHFQ score	−0.871	<.001
Change in LVEDV	−0.278	.179
Change in LVESV	−0.699	<.001
Change in PSD	−0.815	<.001
Change in PHB	−0.725	<.001

*Abbreviations:* LVEDV, left ventricular end-diastolic volume; LVESV, left ventricular end-systolic volume; MLHFQ, Minnesota Living with Heart Failure Questionnaire; PHB, phase histogram bandwidth; PSD, phase standard deviation.

**Table 4:** Univariate Analysis for Predictors of Cardiac Resynchronization Therapy Responders

	B	SE	Wald	*P* value	OR	95% CI for OR
Sex	1.792	0.950	3.556	.059	6.000	0.932–38.629
Non-ischemic cardiomyopathy	1.859	0.904	4.229	.040	6.417	1.091–37.735
Baseline NYHA	−2.277	0.997	5.219	.022	0.103	0.015–0.724
Baseline LVEDV	−0.019	0.008	5.574	.018	0.981	0.965–0.997
Baseline LVESV	−0.021	0.009	5.419	.020	0.979	0.962–0.997
Baseline PSD	0.334	0.130	6.589	.010	1.396	1.082–1.801
Baseline PHB	0.093	0.036	6.481	.011	1.097	1.022–1.178
Perfusion defect	−0.322	0.141	5.178	.023	0.725	0.549–0.956
Lead position concordance with scar	−2.398	0.983	5.953	.015	0.091	0.013–0.624
Lead position concordance with latest activation	1.792	0.913	3.852	.050	6.000	1.003–35.908

*Abbreviations:* B, unstandardized beta coefficient; CI, confidence interval; CRT, cardiac resynchronization therapy; LVESV, left ventricular end-systolic volume; NYHA, New York Heart Association; OR, odds ratio; PHB, phase histogram bandwidth; PSD, phase standard deviation; SE, standard error.

**Table 5:** Receiver Operating Characteristic Curve–derived Cutoff Values for Phase Standard Deviation and Phase Histogram Bandwidth for the Prediction of Response to Cardiac Resynchronization Therapy

Parameter	AUC	Cutoff Values	Sensitivity	Specificity	PPV	NPV
PSD	0.913	>33	86.67	90.00	92.9	81.8
PHB	0.943	>153	100.00	80.00	88.2	100.0

*Abbreviations:* AUC, area under the receiver operating characteristic curve; PPV, positive predictive value; NPV, negative predictive value.
